# Inorganic/Biopolymers Hybrid Hydrogels Dual Cross-Linked for Bone Tissue Regeneration

**DOI:** 10.3390/gels8120762

**Published:** 2022-11-23

**Authors:** Alexandra I. Cernencu, Andreea I. Dinu, Sorina Dinescu, Roxana Trușcă, Mircea Istodorescu, Adriana Lungu, Izabela C. Stancu, Horia Iovu

**Affiliations:** 1 Advanced Polymer Materials Group, University Politehnica of Bucharest, 060042 București, Romania; 2 Department of Biochemistry and Molecular Biology, University of Bucharest, 050095 București, Romania; 3 Research Institute of the University of Bucharest, University of Bucharest, 05066 București, Romania; 4 University Politehnica of Bucharest, 060042 București, Romania; 5 S.C. Medical Ortovit SRL, 011098 București, Romania

**Keywords:** nanostructures, polymer–matrix composites, 3-dimensional reinforcement

## Abstract

In tissue engineering, the potential of re-growing new tissue has been considered, however, developments towards such clinical and commercial outcomes have been modest. One of the most important elements here is the selection of a biomaterial that serves as a “scaffold” for the regeneration process. Herein, we designed hydrogels composed of two biocompatible natural polymers, namely gelatin with photopolymerizable functionalities and a pectin derivative amenable to direct protein conjugation. Aiming to design biomimetic hydrogels for bone regeneration, this study proposes double-reinforcement by way of inorganic/biopolymer hybrid filling composed of Si-based compounds and cellulose nanofibers. To attain networks with high flexibility and elastic modulus, a double-crosslinking strategy was envisioned—photochemical and enzyme-mediated conjugation reactions. The dual cross-linked procedure will generate intra- and intermolecular interactions between the protein and polysaccharide and might be a resourceful strategy to develop innovative scaffolding materials.

## 1. Introduction

Tissue engineering has evolved because of research aiming to find tissue analogues of biologically inspired design to restore or improve the functionality of a damaged tissue. Particularly, bone tissue engineering (BTE) has advanced tremendously since the first efforts to restore compromised bone using synthetic materials based on calcium phosphates, and is now focused on preparing materials that function as a framework for the adhesion of cells and mineralization, transiently replacing the extracellular matrix (ECM) [1,2,3].

Due to their inherent quality to simulate multiple functionalities of the natural ECM, hydrogels have surfaced as a candidate of choice for designing scaffolds capable of providing an optimum microhabitat to facilitate the in vivo remodeling of bone tissue [4,5]. Natural hydrogels have a lower immunological response than synthetic hydrogels and possess several sites where cell-interactive signals or other functional groups can be added to modify their backbone [6,7]. Specifically, gelatin-based scaffolds have piqued researchers’ interest and have been thoroughly investigated for numerous biomedical applications on account of their superior biocompatibility, simplicity of processing and cost-effective availability [8,9,10]. To actively control their mechanical performance and degradation rate and to eliminate the requirement of toxic crosslinking agents, methacrylate groups are desirably grafted onto gelatin backbones, yielding photocrosslinked hydrogels based on gelatin methacryloyl (GelMA) [11,12,13,14,15]. Furthermore, gelatin and its derivatives are also ideal candidates to apply biological crosslinking strategies using various enzymes such as naturally occurring transglutaminases (TGs) that mediate the formation of isopeptide bonds [16,17,18]. Both mammalian and microbial TG have been commonly used for decades as a functional ingredient in food-processing industries to crosslink different kinds of proteinous polymeric materials of animal or plant origin (meat, milk, whey or soy products) to obtain improved features [19,20,21,22,23,24]. This strategy subsequently encouraged new crosslinking approaches for various peptides and/or proteins to generate scaffolds for tissue regeneration [25,26,27,28]. An increasingly versatile approach to achieve more cohesive networks based on GelMA is to use TG crosslinking in conjunction with photo-polymerization. Recent research which evolved around this concept has received considerable attention for cell-laden hydrogels [29] or to fabricate scaffolds through 3D bio-printing [30,31] and has brought fresh windows of opportunity in the biomedical field.

The transamidation reactions mediated by TGs also represented an inspiration source for the in situ crosslinking of non-proteinaceous molecules and further for designing materials with various applications [32,33,34,35,36,37,38,39]. Highly relevant polysaccharides such as hyaluronic acid [40,41,42], pectin or alginate [43,44] were designed and specifically chemically modified to act as effective TG substrates. In the context of mimicking the cellular microenvironment, protein–polysaccharide complex systems, including TG-mediated-conjugates, have been proposed as an important strategy for tissue development due to the excellent juxtaposition of the structural and biological features offered by each component [45,46,47,48,49,50,51].

In addition to combining proteins with polysaccharides, hybrid nanocomposites are also considered for the development of synthetic BTE materials [52,53,54,55]. Among all of the best options for inorganic nano-reinforcement, aside from commonly used calcium phosphate ceramics, silica nanoparticles are gaining popularity as a promising mineral phase that can promote bone formation [56,57,58,59]. Among Si-based compounds, polyhedral oligomeric silsesquioxanes (POSSs) represent a special family of 0D nanoparticles that have recently received considerable attention. Considering the functionalities envisioned at silica cage corners (1–3 nm), POSSs can be designed to act either as nano-filler or as a crosslinking agent enabling chemical interactions with the macromolecular chains to prevent aggregation [60,61,62,63,64,65]. The most recent advances launched the idea of dual-filler nanocomposites to increase the coherence between the organic matrix and the discontinuous phase and further achieve enhanced final properties. This approach was also studied in the context of modifying carbon nanofibers with functionalized POSS nanoparticles using carbodiimide chemistry to obtain new hybrid functional nanocomposites with various applications [66]. Moreover, POSS-functionalized carbon fibers embedded in polylactic acid for bone tissue regeneration were also envisioned and exhibited great mineralization properties [67]. Upon these grounds, and considering the approach of using materials that are readily available from non-toxic bio-resources, it is particularly interesting to use cellulose nanofibers (CNFs) as the secondary nanostructured compound to homogeneously disperse various types of nanostructures in aqueous media and prevent their reagglomeration [68,69,70,71,72,73]. Such a synergistic effect is fully compatible with the current surge of interest in CNF in tissue engineering, given the average diameter of the fibers (5–30 nm), which is comparable to the thickness of natural ECM fibers [74,75,76]. The combination of hydrogels and different dimensional nanoparticles has been shown to be one of the most appealing solutions for generating novel bone tissue structures [77].

In this view, the current research expands on the concept of double-reinforced hydrogels, presenting for the first time the potential prospect of combining POSSs and CNFs as reinforcing agents. With another focus on the effect of POSS side functionalities, two distinct types of inorganic nano-reinforcing agents were used: non-functionalized (POSS0) and 1-methacrylated (POSS1). To design novel hydrogel nanocomposites, this study focusses on photo-crosslinked organic networks based on GelMA and a pectin derivative (amidated pectin, AP). In addition to the prevalent chemical crosslinking, a TG-based chemo-enzymatic approach was also employed to prepare GelMA and AP conjugates and to promote protein–polysaccharide covalent bonds. To evaluate the feasibility of double-reinforced hydrogels, we characterized their swelling, mechanical and morphological properties. In addition, material–cell interactions were investigated through in vitro assays. To the best of our knowledge, no research study has previously been published concerning the usage of dual nanocomposites based on hybrid hydrogels with desirable biological features able to facilitate early mineralization. The newly developed multicomponent materials qualified to faithfully mimic native ECMs may open new avenues in the context of developing bone regeneration materials.

## 2. Results and Discussion

### 2.1. Structural Evaluation of the Nanocomposite Hydrogels by Fourier Transform Infrared (FTIR) Spectroscopy

**Figure 1:**
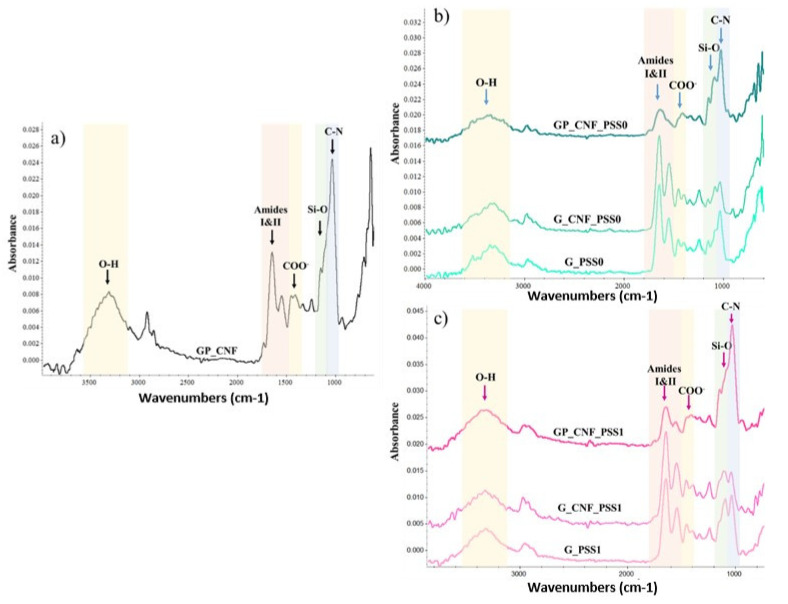
FTIR spectra of (**a**) GP_CNF; (**b**) POSS0-reinforced and (**c**) POSS1-reinforced hydrogels.

The nanocomposite hydrogels were analyzed using FTIR spectrometry to examine the potential interactions between the constituent materials. The FTIR spectra depicted in Figure 1a–c present the main absorption bands that correspond to the precursor polymers and nano-reinforcing agents. Hence, all the spectra exhibited specific signals in the 1700–1600 cm^−1^ and 1590–1500 cm^−1^ regions attributed to amide I and II, respectively, from GelMA [78]. The presence of CNFs is highlighted by the presence of peaks at approximately 1430 cm^−1^ and 3300 cm^−1^ assigned to the symmetrical deformation of the -COO− and to the stretching of -OH, respectively [79,80]. The successful incorporation of POSS molecules is confirmed by the characteristic absorption bands in the form of a shoulder (1080–1090 cm^−1^) for all the POSS-nano-reinforced hydrogels [81]. Furthermore, considering the interactions that occur during crosslinking, it was observed that the peaks appeared at around 1020 cm^−1^, which could be attributed to the C-N stretching vibration as the process of enzymatic crosslinking of GelMA is based on isopeptide formation [31]. This peak appears to be clearly defined for the AP-containing samples, probably due to its contribution in the enzymatic crosslinking.

### 2.2. Investigation of Mechanical Behavior under Compressive Stress

**Figure 2:**
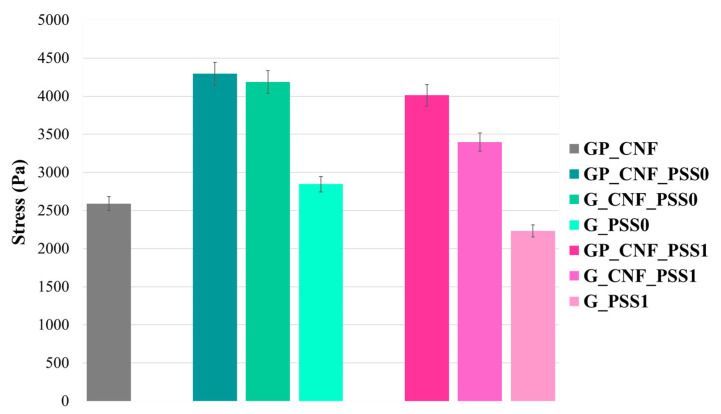
Comparative results of compressive mechanical tests.

The mechanical performance exhibited by the newly synthesized nanocomposite hydrogels was evaluated using uniaxial compression investigations. The amount of stress needed to impart a 5% deformation in the proposed nanocomposite hydrogels is depicted in Figure 2. The findings revealed that the composition, as well as the type of POSS, have a significant effect the material’s resistance to applied stress.

Herein, the use of a more complex polymeric mixture increases the mechanical resistance due to the development of a stiffer network with a higher cross-linking density enabled by the simultaneous use of photopolymerization and the enzymatic crosslinking of both GelMA and AP. To achieve the desired mechanical properties, it is evident that simply including the polysaccharide as a structural component will not be sufficient. Hereby, the incorporation of nanostructured components brings a significant contribution to the materials’ compressive resistance. Specifically, the dual-filler nanocomposites (GP_CNF_PSS0 and GP_CNF_PSS1) are able to withstand a compressive stress of 35–40% higher than the control GP_CNF. It can be observed that POSS0 samples revealed a slightly higher resistance to deformation compared to the POSS1-reinforced hydrogels. The reinforcement effect is probably induced by the dispersion and dissipation of stress across the polymeric matrix due to the interactions between the macromolecular chains and nanosized materials [54,61,62]. In addition, CNF acts as a physical boundary to keep the POSS fillers from re-agglomerating. The nano-reinforcement of hydrogels with CNF was employed to enhance the compressive resistance of hydrogels. As a point of comparison, the values of the stress needed to impart a 5% deformation to demonstrate that the samples containing CNF were able to resist to approximately 30% higher compressive force than the hydrogels without CNF. The presence of CNF serves as structural reinforcement by constructing a more elastic network inside the hydrophilic matrix, and this nanomaterial was acknowledged for its ability to improve the mechanical properties of weak networks [82,83,84].

The samples that exhibited the least compressive resistance were the nanocomposite hydrogels based on GelMA and POSS. As GelMA forms brittle hydrogels that do not present satisfactory mechanical resistance for load-bearing applications in tissue engineering, such as bone scaffolds [85], it was expected that G_PSS0 and G_PSS1 express the lowest mechanical resistance. In this instance, POSS nanoparticles cluster in the lack of CNF, whereas the fragile nature of the protein chains breaks easier in the absence of pectin.

### 2.3. Scanning Electron Microscopy (SEM) Imaging

**Figure 3:**
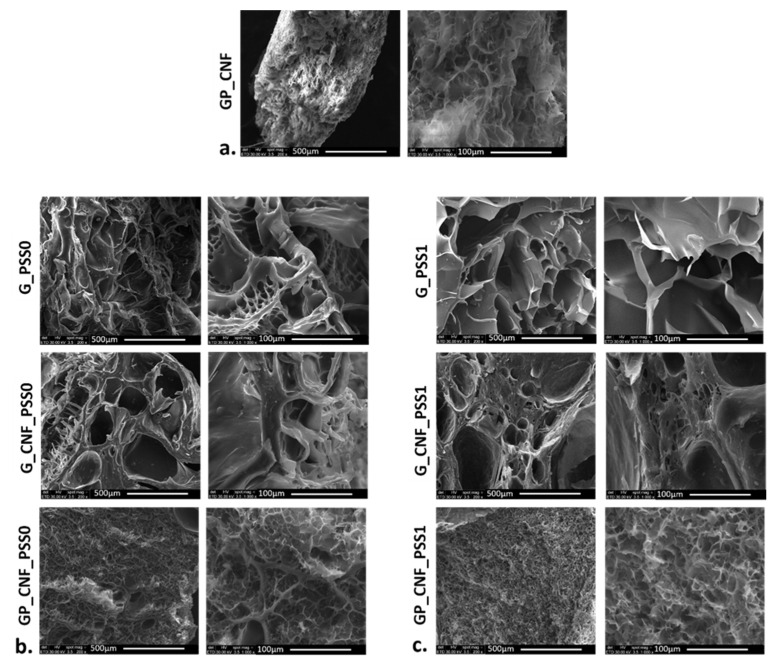
Cross-sectional SEM micrographs showing two different magnifications (200× and 1000×) of the morphology for (**a**) GP_CNF; (**b**) PSS0-enriched; and (**c**) PSS1-enriched scaffolds, respectively, (scale bars 500 μm and 100 μm).

Given the vital role of microstructure in scaffold performance, the characterization of the internal morphology is essential: the SEM technique was employed to acquire insightful images of the studied nanocomposite materials. Overall, the hydrogels’ internal architecture is characterized by a large number of micropores of varying diameters that were randomly dispersed throughout the freeze-dried samples [86] (Figure 3).

The observed morphology at 200× magnification for G_PSS0 and G_PSS1 samples reveals numerous pores with dimensions ranging from 50 to 500 μm. It seems that these pores were not interconnected, but arranged in separated pockets delimitated by fine walls inside the hydrogel [87]. Increasing the magnification at 1000×, a distinct type of smaller pores can be observed; these pores seem to be separated by rougher struts and are arranged in a honeycomb pattern [61]. Clusters of POSS nanoparticles are visible, especially in the case of G_PSS0 nanocomposites.

The G_CNF_PSS0 and G_CNF_PSS1 hydrogels exhibited a porous internal architecture as well, with open interconnected pores with diameters varying from 50 to 600 μm (Figure 3b,c). Nevertheless, it is visible that a distinctive morphology from the previous samples was generated due to the presence of CNFs [82].

As porosity is one of the critical requirements that dictate cell adhesion and migration inside the polymeric matrix [88], it was observed that the scaffold density visually increases when increasing the dry content. Given the multi-component system and double-crosslinking approach, GP_CNF_PSS0 and GP_CNF_PSS1 and GP_CNF exhibit more compact structures with numerous smaller micropores with a diameter below 20 μm. Moreover, it appears that the addition of CNF significantly improves POSS dispersion as no clusters can be observed in CNF-reinforced samples [70].

### 2.4. Swelling Behavior

**Figure 4:**
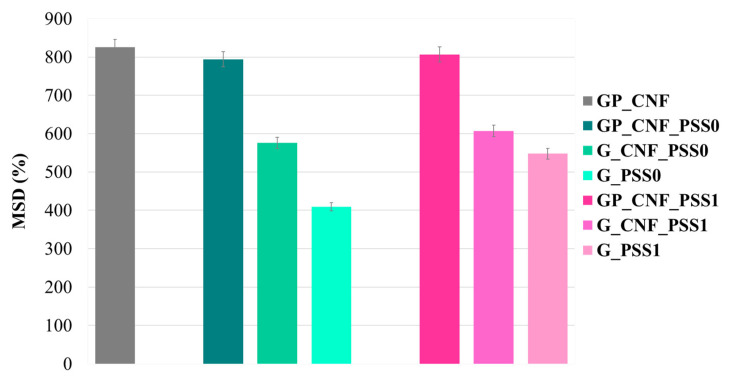
Maximum swelling degree (MSD) of the nanocomposite hydrogels presented comparatively depending on POSS functionalization.

Controlling water absorption is critical for scaffolds with applications in tissue regeneration because it allows the diffusion of nutrients and metabolites into the polymeric matrix and hydrolytic degradation. The hydrophilicity and swelling behavior of the scaffolds were studied herein, taking into consideration both the polymeric composition and the influence of the dual-fillers (Figure 4). The results reveal a strong correlation between sample composition and swelling behavior.

It can be observed that the samples that contain GelMA, AP and CNF hold the greatest fluid uptake capacity (MSD% at 24 h is 825% ± 27), mainly as a result of the excellent hydrophilicity of pectin [89]. Additionally, due to the capacity of CNF to absorb up to 90 times their own weight, the inclusion of nanofibers into the protein network boosts the hydrogel’s swelling potential by creating more porous constructions that allow water penetration [80]. In addition, CNFs are known to form strong hydrogen bonds that modulate the entrance of PBS inside the matrix, so that the incorporation of nanofibers in GelMA hydrogels prevents the early collapse and degradation of the polymeric network due to the rapid infiltration of swelling media [83,90]. Therefore, the single-reinforced hydrogels (GP_CNF) display the higher swelling capacity, proving to have networks with the highest flexibility.

The addition of POSS inorganic reinforcement showed a slight decrease in MSD values, however, it does not have a significant influence on the overall swelling capacity of the materials. Regarding the type of POSS used, it seems that the addition of the inorganic compounds does not exhibit a negative impact on the hydration degree. There are no major differences between the MSD values of GP_CNF_PSS0 (794% ± 23) and GP_CNF_PSS1 (806% ± 19) when compared to their corresponding reference sample. This result can be explained by the synergy of two factors: the considerable hydrophilicity of the used polymers and a complex interconnected and open-pore network that allows the penetration of PBS.

The blank control samples further confirm that the addition of CNF seemed to increase the MSD up to 25%, while the minimum value of MSD was registered for G_PSS1 and G_PSS0 samples.

### 2.5. In Vitro Cytocompatibility Live/Dead Fluorescence Microscopy Assay

**Figure 5:**
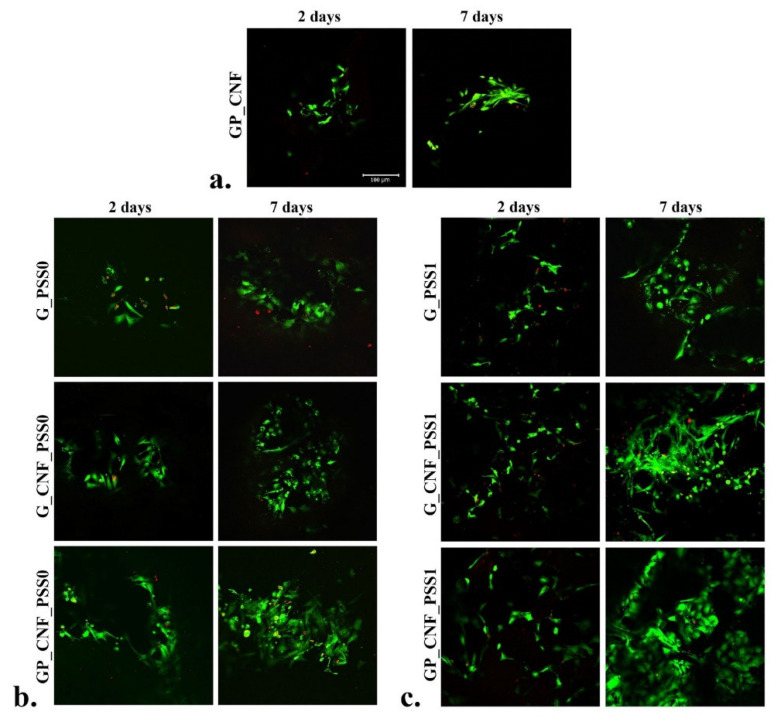
Qualitative evaluation of cell viability and proliferation during seven days of culture under standard conditions observed by Live/Dead fluorescent staining coupled with confocal microscopy in (**a**), GP_CNF scaffold, (**b**) PSS0-enriched scaffolds and (**c**) PSS1-enriched scaffolds. The nuclei of viable cells are green, while the nuclei of dead cells are red. Scale bar 100 μm.

An efficient scaffold for tissue regeneration requires an understanding of how cells behave and respond, as well as an understanding of how the scaffold architecture affects cell survival and adherence. Live/dead fluorescent staining coupled with confocal microscopy (Figure 5) indicated a high amount of live cells in contact with the 3D porous scaffolds two days after cell seeding, suggesting that all materials support cell growth and viability. An improvement in cell adhesion and group formation was observed in contact with POSS-containing materials, suggesting that these scaffolds offer optimal conditions for murine preosteoblast growth.

After seven days, a higher proliferation rate was observed in contact with the G_PSS1 scaffold compared to the G_PSS0 scaffold, indicating that POSS1 offers more favorable conditions for cellular activity. Notably, between day 2 and 7 of culture under standard conditions, the cells proliferated on all tested scaffolds, especially on those enriched with CNF and AP. Moreover, the association between POSS, CNF and AP positively influenced cell proliferation and the formation of larger groups, proving that the prepared dual-filled nanocomposites (GP_CNF_PSS0 and GP_CNF_PSS1) represent feasible candidates for tissue engineering. Apart from the polymeric builders’ biocompatibility, the scaffolds exhibit different morphologies with a highly porous architecture, which has a substantial effect on the cellular response. On these grounds, it was observed that the more complex samples, which include all four major components, demonstrated an increased viability and proliferation of the preosteoblastic cells (Figure 5).

### 2.6. Quantitative Evaluation of Cell Viability, Proliferation and Cytotoxicity

**Figure 6:**
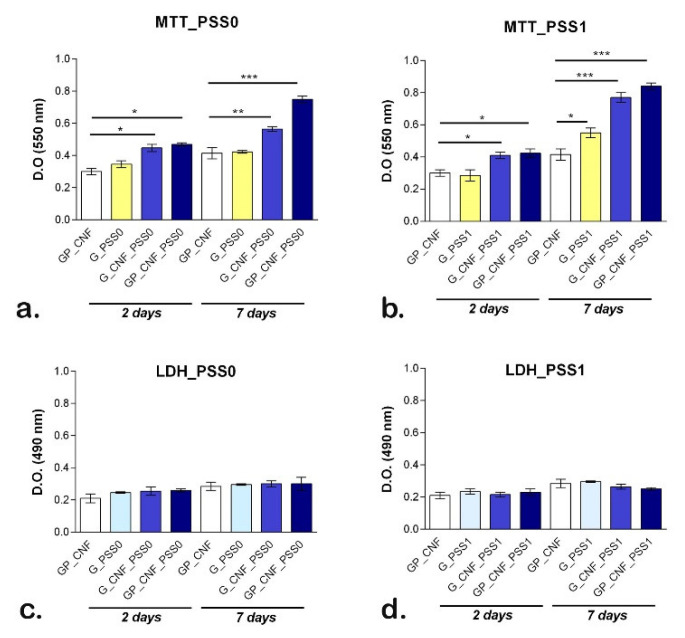
Assessment of biocompatibility of POSS0- and POSS1-enriched scaffolds seeded with murine preosteoblasts. (**a**) MTT assay results regarding MC3T3-E1 cells viability and proliferation after two and seven days of interaction with scaffolds reinforced with POSS0. Statistical significance: * *p* < 0.05, ** *p* < 0.01, *** *p* < 0.001. (**b**) MTT assay results regarding MC3T3-E1 cell viability and proliferation after two and seven days of interaction with scaffolds reinforced with POSS1. Statistical significance: * *p* < 0.05, *** *p* < 0.001. (**c**) LDH assay results regarding the cytotoxicity of scaffolds reinforced with POSS0 in contact with MC3T3-E1 cells, after two and seven days of culture under standard conditions. (**d**) LDH assay results regarding the cytotoxicity of scaffolds reinforced with POSS1 in contact with MC3T3-E1 cells, after two and seven days of culture under standard conditions. The fabrication technique allowed for the generation of porous and flexible scaffolds that showed to facilitate the morphogenesis. The porous internal architecture of the composites enables the ingress of water and nutrients into the scaffold, resulting in increased cell growth and proliferation. Addition of polysaccharides either as a secondary polymer matrix or as reinforcing agent enhanced protein resistance to degradation and maintained the storage modulus of gelatin, while POSS-reinforcement endorsed cell integration and improved scaffold strength.

The quantitative MTT and LDH assay confirmed the results obtained by the Live/Dead assay. Cell viability assessment (Figure 6a) revealed that the cells maintained a good viability rate at two days post-seeding and significant changes (*p* < 0.05) between CNF_PSS0-containing scaffolds and the GP_CNF control were registered. After seven days of culture, significantly increased viability rates (*p* < 0.01) were observed on G_CNF_PSS0 and GP_CNF_PSS0 as compared to the control, while the lowest viability rate was registered on a G_PSS0 scaffold. Between day 2 and 7 of culture, notable differences in cell proliferation were observed in contact with GP_CNF_PSS0.

Additionally, POSS1-containing scaffolds indicate the same ascending viability profile as POSS0-enriched scaffolds (Figure 6b). The metabolic activity of the cells were significantly increased upon two days of incubation (*p* < 0.05) for G_CNF_PSS1 and GP_CNF_PSS1 as compared to the control. Significant differences (*p* < 0.05) between GP_CNF and G_PSS1 were observed after a week of culture, suggesting that POSS1 embedding into scaffolds’ composition provided favorable conditions for cell growth. Furthermore, a significant increase (*p* < 0.001) was registered between the G_CNF_PSS1, GP_CNF_PSS1, and GP_CNF control, indicating that the addition of CNF and AP positively influenced the cell viability [71]; during one week of culture, murine preosteblasts had an increased proliferation profile in contact with POSS1-reinforced scaffolds.

The cytotoxic effect of the 3D porous scaffolds was assessed employing an LDH test. Both POSS0- and POSS1-containing materials exhibit a low cytotoxicity profile, with no significant changes between all scaffolds (Figure 6c,d). The obtained results indicate that CNF, AP and POSS-containing scaffolds do not induce significant toxicity on preosteoblastic cells during 7 days of culture under standard conditions.

### 2.7. Cytoskeleton Development

**Figure 7:**
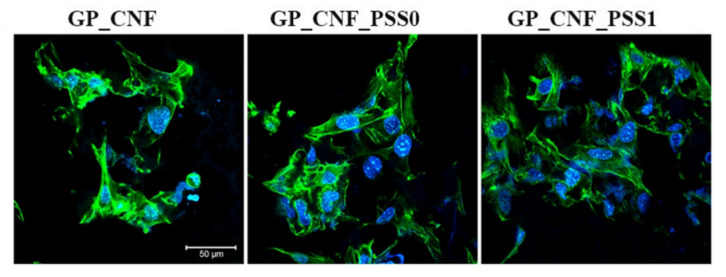
Cytoskeleton developed by murine preosteoblasts cultivated in contact with GP_CNF, GP_CNF_PSS0 and GP_CNF_PSS1 during 48 h of culture under standard conditions, as revealed by phalloidin-FITC staining coupled with confocal microscopy. The nuclei of cells are stained in blue (Hoechst 33342). Scale bar 50 μm.

The development of F-actin filaments confirmed the favorable effects of natural components (CNF and AP) from the scaffolds’ composition as well as the differences in cellular behavior due to the presence of POSS0 and especially POSS1 (Figure 7). The association between protein–polysaccharide conjugates and CNF proved positive influence on cell adhesion and cytoskeleton expansion, considering the presence of large cell groups and elongated F-actin filaments. Furthermore, the POSS0 and POSS1 presence influenced the number of adhered cells and the size of cell groups, since there are considerably more cells adhered on the GP_CNF_PSS0 and GP_CNF_PSS1 in contrast with the GP_CNF control scaffold. Hence, the most remarkable cytoskeleton development was identified in contact with GP_CNF_PSS1 scaffold, suggesting that POSS1 addition is more beneficial for cellular activity as compared to POSS0.

## 3. Conclusions

Double-reinforced hydrogels are explored in this study, with POSS and CNF being used as reinforcing agents for the first time. Two separate forms of inorganic nano-reinforcing agents were employed, with an eye on the impact of POSS side functionalities. Based on GelMA and a pectin derivative, photo-crosslinked organic networks were generated to build new hydrogel nanocomposites in this research (amidated pectin, AP). This strategy combined with enzymatic crosslinking was used to generate GelMA and AP conjugates and increase protein–polysaccharide covalent connections, in addition to the more common chemical crosslinking technique. Newly synthesized hydrogel nanocomposites show improved mechanical performance due to the formation of a stiffer network with a higher crosslinking density facilitated by the simultaneous use of photopolymerization and the enzymatic crosslinking of both GelMA and AP, according to the results of mechanical testing. The type of POSS used had a considerable influence on the material’s ability to withstand stress, where POSS0 samples exhibited greater deformation resistance than POSS1-reinforced hydrogel samples. The reinforcing effect is most likely caused by the dispersion and dissipation of stress within the polymeric matrix as a result of interactions between the organic chains and nanoparticles. The hydrophilic and swelling characteristics of the multicomponent hydrogels were evaluated, and it was discovered that the single reinforced hydrogels (GP_CNF) had the greatest swelling capacity, indicating that they have the most flexible networks. Here, the inclusion of POSS inorganic reinforcement resulted in a modest drop in MSD values but had no meaningful effect on the materials’ total swelling capacity. A very porous structure is present in the scaffolds, which results in a wide range of architectures and a significant influence on the cellular response. Porous and flexible scaffolds were developed using the double-crosslinking approach and it was shown to be beneficial in the process of cell growth. The association between POSS, CNF and AP was found to have a beneficial influence on cell spread and the development of larger groups, demonstrating that the dual-filled nanocomposites (GP_CNF_PSS0 and GP_CNF_PSS1) developed in this study are viable candidates for tissue engineering applications. Aside from that, it was determined that the presence of POSS0 and POSS1 influenced how many cells attached to the scaffolds and the size of cell groups, as there are much more cells adherent to the POSS0 and POSS1 scaffolds compared to the GP_CNF control scaffold. GP_CNF_PSS1 scaffold, on the other hand, has been shown to promote the most spectacular cytoskeleton formation, demonstrating that the inclusion of POSS1 is more favorable to cellular activity when compared to the addition of POSS0.

## 4. Materials and Methods

### 4.1. Materials

Methacrylated gelatin (GelMA) was obtained by chemically modifying Type B gelatin from bovine skin (Sigma Aldrich, Darmstadt, Germany) with methacrylic anhydride following a protocol described in a previous work [90]. The degree of substitution of 33% was evaluated using ^1^H-NMR spectroscopy as previously described in [90]. A commercial amidated pectin of food grade, GRINDSTED^®^ LA 410 extracted from citrus peel (Danisco, Czech Republic) was kindly supplied by KUK Romania and used without any further purification. With a degree of esterification of 31% and a degree of amidation of 19%, this food additive (E440ii) is considered a lowly methoxylated amidated pectin. Carboxylated-cellulose nanofibrils (CNFs) were synthesized using a TEMPO-mediated oxidation of bleached softwood kraft pulp (kindly offered by StoraEnsoTM), as disclosed in our earlier work [79]. CNFs were obtained as a gel-like suspension with a solid content of 1.2% and a degree of modification of 835 µmol/g evaluated through conductometric titration [91]. PSS-Octakis(dimethylsilyloxy) substituted (POSS0), PSS-(1-Propylmethacrylate)-Heptaisobutyl substituted (POSS1) and the photo-initiator Irgacure 2959 were bought from Sigma Aldrich and used without further purification. Microbial TGs (food grade) with an enzyme activity of 80–120 U/g were donated by RAPS Romania and used as received. Phosphate buffer saline (PBS) tablets yielding 10 mM phosphate, 2.7 mM KCl and 0.14 M NaCl, pH 7.4, at 25 °C were purchased from Carl Roth. MC3T3-E1 cell line was purchased from ATCC, Manassas, FA, USA. Dulbecco’s modified Eagle medium (DMEM), antibiotic–anti-mycotic solution, 3-(4,5 dimethylthiazolyl-2)-2,5-diphenyltetrazolium bromide (MTT) reagent for a quantitative evaluation of cell viability, TOX7-KT lactate dehydrogenase (LDH) assay kit for evaluation of materials’ cytotoxicity, bovine serum albumin (BSA), Triton-X100, paraformaldehyde (PFA) solution, phalloidin-FITC and Hoechst 33342 solution for fluorescence staining of F-actin filaments and cell nuclei were purchased from Sigma-Aldrich, Germany. Fetal bovine serum (FBS) and Live/Dead Viability/Cytotoxicity Kit for mammalian cells were purchased from Thermo Fisher Scientific, St. Bend, OR, USA. All the chemicals were purchased of reagent grade and were used as received without further purification.

### 4.2. Preparation of the Hydrogel Nanocomposites

To prepare the multicomponent hydrogels, the appropriate amount of POSS was dispersed in CNF suspension (1.2% *w*/*v*) to obtain a 0.5% *w*/*w* concentration of inorganic nanoparticles within the nanofibers suspension. Next, GelMA (10% *w*/*w*) was included into the mixture and allowed to dissolve under magnetic stirring at 40 °C. Then, an appropriate amount corresponding to 2.5% *w*/*w* of AP was added and stirred at 40 °C for 15 min. Targeting both photo- and enzymatic crosslinking, Irgacure 2959 and the TG enzyme were both included into each mixture at concentrations of 0.1% *w*/*w* and 5 U/mL, respectively. The polymeric mixtures were poured into Petri dishes and subjected to UV radiation (365 nm) for 5 min on every side and then left for 24 h at 37 °C to accomplish the enzyme-driven crosslinking mechanism. According to the type of the POSS used (POSS0 or POSS1), the as-prepared samples were further denoted as GP_CNF_PSS0 and GP_CNF_PSS1.

Additionally, to evaluate the impact of certain component(s) within the complex nanocomposite hydrogels, three main types of control samples were simultaneously prepared to validate the experiments from the present research study. Hence, the previously described protocol was employed, maintaining the GelMA matrix and double crosslinking, and further adapted in accordance with the investigated effect. (a) To evaluate the POSS impact, a control sample was obtained using the protein–polysaccharide conjugates as a polymeric matrix and CNF was employed as a single reinforcement (GP_CNF). (b) To establish the effect of the polysaccharide (AP), two reference samples using only the proteinaceous matrix were obtained with both POSS and CNF nanofillers embedded within (G_CNF_PSS0; G_CNF_PSS1). (c) Additionally, simple POSS-filled GelMA networks (G_PSS0; G_PSS1) were obtained and used as experimental negative controls.

**Figure 8:**
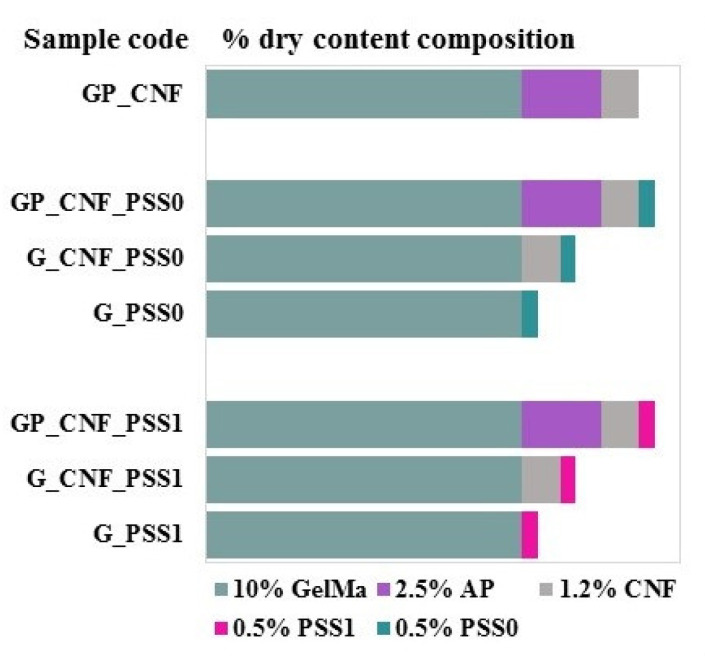
Schematic diagram depicting the nanocomposite hydrogel composition.

All the prepared specimens were rinsed thoroughly with distilled water to remove any unbound or loosely attached reagents and were then freeze-dried. The composition of each sample with respect to the dry content together with the sample codes are described in Figure 8.

### 4.3. Evaluation of Structural Properties of the Nanocomposite Hydrogels by Fourier Transform Infrared (FTIR) Spectroscopy

The proposed multicomponent hydrogels were characterized from the structural point of view by Fourier Transform Infrared (FTIR) spectrometry using a Vertex 70 Bruker FTIR equipment in the attenuated total reflectance (ATR) mode. The analysis was carried out with 4 cm^−1^ resolution by performing 32 scans between 600 and 4000 cm^−1^ at ambient temperature.

### 4.4. Investigation of Mechanical Behavior under Compressive Stress

Uniaxial compression tests were performed on three cylindrical hydrated samples of each composition of the nanocomposite hydrogel. The equipment used was CT3 texture analyzer (Brookfield) assembled with a cell weighing 4500 g and a TA4/100 compression accessory, the tests being operated at room temperature up to deformations of 2% and 5% with a rate of compression of 0.5 mm/s. The tests were repeated three times under identical conditions and the average values were reported.

### 4.5. Scanning Electron Microscopy (SEM) Imaging

The morphology of the obtained scaffolds was observed using a high-resolution FEI Quanta Inspect F50 microscope with a field emission gun (SEM, 1.2 nm resolution). The images were obtained by recording the secondary electron beam resulted, with an energy of 30 keV. To visualize the internal structure of the scaffolds, cross-sections were prepared from freeze-dried samples. The so-prepared specimens were coated with gold using a sputter coating machine before being placing into the SEM chamber.

### 4.6. Swelling Behavior

To evaluate the swelling behavior in PBS, samples of each composition were accurately weighed to obtain the dry mass (*m_d_*) and then immersed in PBS at room temperature. The wet weight (*m_s_*) of the specimens was periodically measured by removing the samples from the PBS, gently blotting with filer paper, and immediately weighing them. The maximum swelling degree (*MSD*) was recorded at 24 h and calculated using Equation (1):

(1) $$MSD\;\left(\%\right)=\frac{m_{s}-m_{d}}{m_{d}}\times 100.$$

The tests were repeated three times under identical conditions and the average values were reported.

### 4.7. In Vitro Cytocompatibility Live/Dead Fluorescence Microscopy Assay

Murine preosteoblastic MC3T3-E1 cells were cultivated under standard conditions (37 °C and 5% CO_2_) in DMEM medium containing 10% FBS and 1% antibiotic–antimycotic solution. The samples were cut as discs of 1 cm diameter subjected to UV light sterilization. The cells were cultured at a 2 × 10^5^ cells/cm^2^ density on the 3D scaffolds and incubated under standard test conditions for seven days, during which the time quantitative and qualitative biocompatibility tests were conducted.

A Live/Dead fluorescent staining kit was used to qualitatively measure the ratio between the viable and dead cells. The staining solution was obtained following the supplier’s instructions and incubated with the specimens for 1 h at room temperature in the dark. The green-labeled viable cells and red-labeled dead cells were examined using a Zeiss 710 confocal microscope, and the images were examined using Zeiss Zen software.

### 4.8. Quantitative Evaluation of Cell Viability, Proliferation and Cytotoxicity

The MTT assay was used to assess the viability and proliferation of murine preosteoblasts in contact with the porous 3D scaffolds. The samples were cultured for 4 h under standard test conditions with a 1 mg/mL MTT solution to allow the crystallization of formazan. The obtained formazan crystals were dissolved in isopropanol, and the solution’s absorbance was recorded at 550 nm with a FlexStation3 Spectrophotometer (Molecular Devices, San Jose, CA, USA). The LDH assay was used to determine the samples’ cytotoxic effects upon mouse preosteoblasts. The reaction mix obtained according to the manufacturer’s guidelines was blended with the culture media from the specimens. The final solution was incubated for 10–15 min at room temperature in the absence of light, and the absorbance of the finished product was recorded using a FlexStation3 Spectrophotometer at 490 nm.

### 4.9. Cytoskeleton Fluorescent Staining

After 48 h of incubation under standard conditions, the adherence of MC3T3-E1 cells to the proposed 3D scaffolds was investigated. A 4% PFA solution was used for fixing the 3D cultures for 1 h. Then, the seeded scaffolds were permeabilized for 20 min in a 0.1% Triton-X100 solution prepared in 2% BSA. A phalloidin-FITC solution was used to stain the F-actin filaments for 1 h. Hoechst 33342 solution was used to stain the nuclei of the cells. The images were taken with a Zeiss 710 confocal microscope and analyzed with Zeiss Zen software.

### 4.10. Statistical Analysis

All the experiments were conducted in triplicate (*n* = 3) and the data were plotted using the GraphPad Prism 6.0 software (GraphPad Software Inc., San Diego, CA, USA). The statistical validity was assessed using a one-way ANOVA technique and the Bonferroni post test, with a statistical difference of *p* < 0.05 considered significant.

## Data Availability

The data presented in this study are available upon request from the corresponding author. Data were obtained as described.
